# Single-leg landing neuromechanical data following load and land height manipulations

**DOI:** 10.1016/j.dib.2016.07.011

**Published:** 2016-07-16

**Authors:** Andrew D. Nordin, Janet S. Dufek

**Affiliations:** a University of Michigan, United States; b University of Nevada, Las Vegas, United States

**Keywords:** Kinematic, Kinetic, Electromyographic, Drop landing

## Abstract

Lower extremity sagittal kinematic and kinetic data are summarized alongside electrical muscle activities during single-leg landing trials completed in contrasting external load and landing height conditions. Nineteen subjects were analyzed during 9 landing trials in each of 6 experimental conditions computed as percentages of subject anthropometrics (bodyweight: BW and subject height: H; BW, BW+12.5%, BW+25%, and H12.5%, H25%). Twelve lower extremity variables (sagittal hip, knee, ankle angles and moments, vertical ground reaction force (GRFz), gluteus maximus, biceps femoris, vastus medials, medial gastrocnemius, and tibialis anterior muscles) were assessed using separate principal component analyses (PCA). Variable trends across conditions were summarized in “Neuromechanical synergies in single-leg landing reveal changes in movement control. *Human Movement Science*” (Nordin and Dufek, 2016) [1], revealing changes in landing biomechanics and movement control.

**Specifications Table**

**Table:** 

Subject area	Kinesiology
More specific subject area	Biomechanics
Type of data	Figures
How data was acquired	Kinematic (10 camera Vicon MX-T40S), kinetic (Kistler force platform, Type 9281CA), and electromyographic (EMG) (Noraxon Myosystem 2000) time series data.
Data format	Filtered, analyzed
Experimental factors	Kinematic data were low-pass filtered (15 Hz cutoff), ground reaction force data were low-pass filtered (50 Hz cutoff), EMG data were band pass filtered (15–300 Hz cutoffs) rectified and low pass filtered (15 Hz cutoff).
Experimental features	Nineteen healthy volunteers were analyzed during 9 single-leg drop landing trials in each of six experimental conditions (3 load and 2 landing height: BW, BW+12.5%, BW+25% and H12.5% and H25%; BW is subject bodyweight and H is subject standing height). Lower extremity sagittal joint angles and moments (hip, knee, ankle), vertical ground reaction force (GRFz), and electrical muscle activities were analyzed in each trial.
Data source location	University of Nevada, Las Vegas, Las Vegas, NV, USA
Data accessibility	All relevant data are presented within the article.

**Value of the data**

• Data include ensemble curves and principal components extracted from single-leg landing trials among 19 healthy volunteers.
• Previously injured or individuals susceptible to injury may reveal different movement patterns following load and landing height manipulations.
• Baseline comparisons may be useful for identifying atypical landing biomechanics.

## 1 Data

The six figures present patterns of change among 12 lower extremity variables collected in a single-leg landing task following external load and landing height manipulations. Ensemble time series plots and principal component analysis (PCA) results are shown for each variable. Principal component (PC) loading vectors and PC scores present magnitude and temporal changes among conditions, along with analysis of variance results (ANOVA, *p*<0.05).

Lower extremity single-leg landing biomechanical data were summarized among subjects for sagittal joint angles and moments (hip, knee, ankle), vertical ground reaction forces (GRFz), and muscle activation patterns (gluteus maximus, biceps femoris, vastus medialis, medial gastrocnemius, tibialis anterior). Subject-specific anthropometrics were used to calculate load and landing height conditions (bodyweight: BW, and subject height: H). Six experimental load and landing height combinations were completed: BW, BW+12.5%, and BW+25% at H12.5% and H25%. Principal component analyses (PCA) were performed for each lower extremity variable. PC loading vectors depicted movement patterns. PC scores summarized movement pattern differences among conditions.

## 2 Experimental design, materials and methods

We analyzed 19 healthy volunteers (15M, 4F, age: 24.3±4.9 y, mass: 78.5±14.7 kg, height: 1.73±0.08 m) during 9 single-leg drop landing trials in each of six experimental conditions. External load and landing height were adjusted as percentages of subject-specific anthropometrics (bodyweight: BW, and subject height: H). We applied external loads to the trunk with small backpacks and iron weights. Landing height was manipulated with an adjustable platform. Conditions were: 1.) BW•H12.5, 2.) BW+12.5•H12.5, 3.) BW+25•H12.5, 4.) BW•H25, 5.) BW+12.5•H25, 6.) BW+25•H25, completed in randomized order.

Subjects completed single leg landing trials on their preferred limb. We collected 12 lower extremity variables: sagittal joint angles and moments (hip, knee, and ankle), vertical ground reaction force (GRFz), and electrical muscle activities (gluteus maximus, biceps femoris, vastus medialis, medial gastrocnemius, and tibialis anterior muscles). Kinematic data were collected with 10-camera system (16-point spatial model; Vicon Plugin-Gait; MX-T40S; 200 Hz), kinetic data were collected with a force platform (Kistler Type 9281CA; 2000 Hz), and electromyographic (EMG) data were collected with passive dual surface electrodes (Ag/AgCl; Noraxon Myosystem 2000; 2000 Hz).

Kinematic data were low-pass filtered (15 Hz cutoff; Matlab R2012a), ground reaction force data were low-pass filtered (50 Hz cutoff), and internal joint moments were computed using matched filter cutoffs (15 Hz). EMG data were band pass filtered (15–300 Hz cutoff), full-wave rectified, and low-pass filtered (15 Hz cutoff). We analyzed data across the landing phase (from GRFz>20 N to the time vertical center of mass velocity reached zero). We expressed joint angles in degrees, joint moments were normalized to bodyweight and subject height (BW•H), and EMG data were normalized to mean dynamic baseline activity recorded in the experimental condition with the lowest task demands (BW•H12.5) [2].

Prior to principal component analyses (PCA), we temporally normalized the landing phase to 101 points using cubic spline interpolation (Matlab R2012a). We then converted each time series waveform to z-scores by subtracting the subject׳s baseline mean and dividing by the baseline standard deviation (BW•H12.5). For each of the 12 lower extremity variables, we assembled 1026×101 dimension matrices (19 subjects×3 loads×2 heights×9 trials=1026; times series length=101). Principal components (PCs) explaining greater than 90% cumulative explained variance (EV) were extracted from the covariance matrix of each variable, and PC scores were computed for each trial and PC (Matlab R2012a). PC scorea means were aggregated for each subject from the 9 analyzed trials and used for inferential testing. Separate 3×2 (load×height) repeated measures analyses of variance (ANOVAs) were conducted for each extracted PC and used in evaluating movement pattern changes across conditions (IBM SPSS Statistics Version 20) [3], [4]. We performed follow-up one-way repeated measures ANOVAs, simple main effects analysis, degree of freedom Huynh-Feldt corrections, and Bonferroni corrections during pairwise comparisons, as necessary [5].

**Fig. 1:**
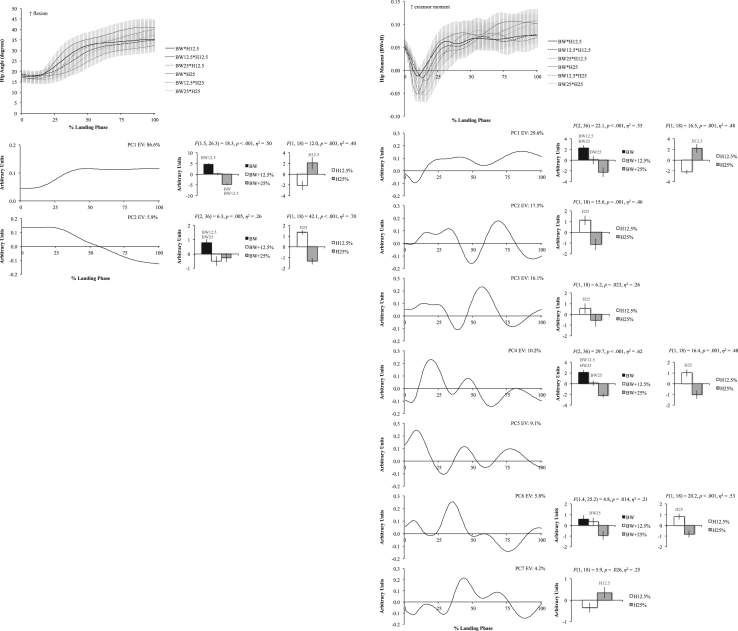
Hip angle (left column) and hip moment (right column) ensemble plots by condition (top row; ±standard deviation) and PCA results. Principal component (PC) loading vectors presented in order of descending explained variance (left; EV) alongside PC score means by condition (right; ±standard error, pairwise comparisons *p*<0.05). BW is bodyweight and H is subject height.

**Fig. 2:**
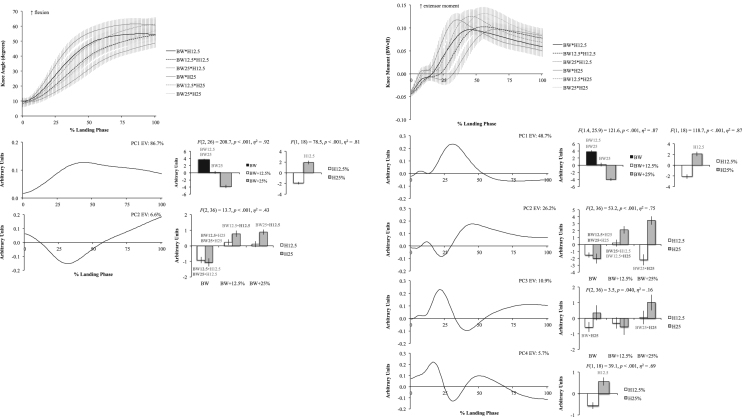
Knee angle (left column) and knee moment (right column) ensemble plots by condition (top row; ±standard deviation) and PCA results. Principal component (PC) loading vectors presented in order of descending explained variance (left; EV) alongside PC score means by condition (right; ±standard error, pairwise comparisons *p*<0.05). BW is bodyweight and H is subject height.

**Fig. 3:**
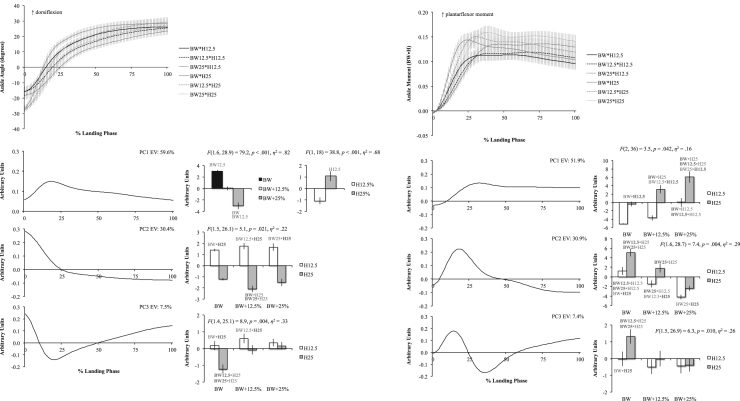
Ankle angle (left column) and ankle moment (right column) ensemble plots by condition (top row; ±standard deviation) and PCA results. Principal component (PC) loading vectors presented in order of descending explained variance (left; EV) alongside PC score means by condition (right; ±standard error, pairwise comparisons *p*<0.05). BW is bodyweight and H is subject height.

**Fig. 4:**
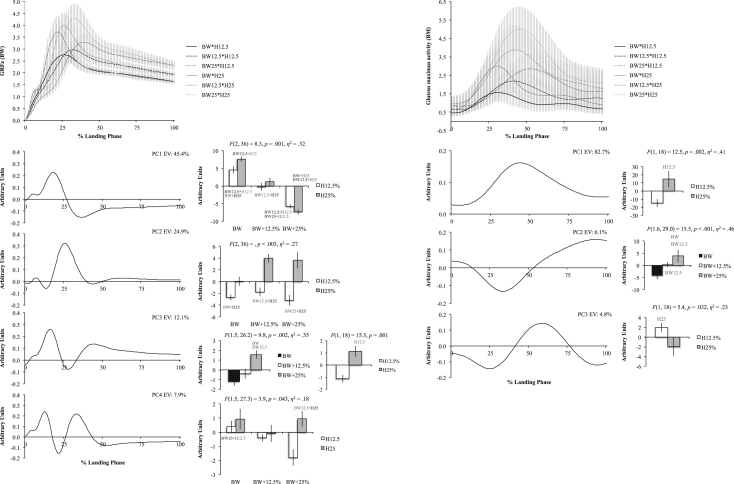
Vertical ground reaction force (GRFz; left column) and gluteus maximus (right column) ensemble plots by condition (top row; ±standard deviation) and PCA results. Principal component (PC) loading vectors presented in order of descending explained variance (left; EV) alongside PC score means by condition (right; ±standard error, pairwise comparisons *p*<0.05). BW is bodyweight, H is subject height, and BM is baseline multiple (BW•H12.5).

**Fig. 5:**
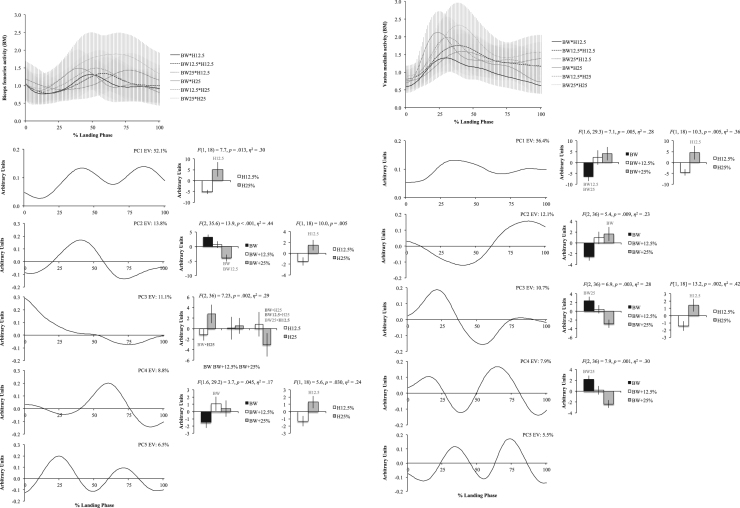
Biceps femoris (left column) and vastus medialis (right column) ensemble plots by condition (top row; ±standard deviation) and PCA results. Principal component (PC) loading vectors presented in order of descending explained variance (left; EV) alongside PC score means by condition (right; ±standard error, pairwise comparisons *p*<0.05). BW is bodyweight, H is subject height, and BM is baseline multiple (BW•H12.5).

**Fig. 6:**
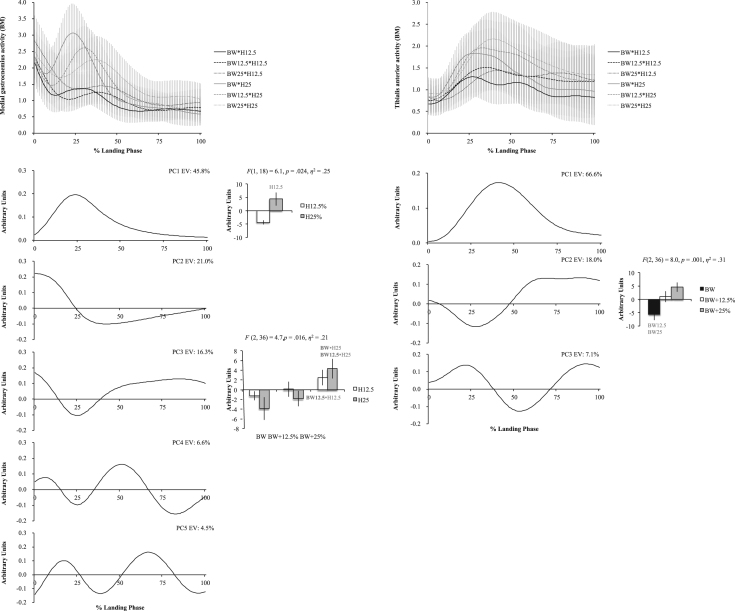
Medial gastrocnemius (left column) and tibialis anterior (right column) ensemble plots by condition (top row; ±standard deviation) and PCA results. Principal component (PC) loading vectors presented in order of descending explained variance (left; EV) alongside PC score means by condition (right; ±standard error, pairwise comparisons *p*<0.05). BW is bodyweight, H is subject height, and BM is baseline multiple (BW•H12.5).

Ensemble curves (aggregated among subjects) were plotted by condition for each lower extremity variable (Fig. 1, Fig. 2, Fig. 3, Fig. 4, Fig. 5, Fig. 6, top). Extracted PCs were plotted below ensemble plots in order of descending EV. PC score means were presented beside each PC loading vector, indicating statistically significant condition differences where appropriate (*α*=0.05). Load and landing height PC score means were presented in each condition following significant interaction. Height PC score means were presented following a significant height main effect and load PC score means were presented following a significant load main effect. In each case, significant ANOVA results were included above PC score means, with significant pairwise comparisons highlighted (*α*=0.05). Hip angles and moments were show in Fig. 1, knee angles and moments were shown in Fig. 2, ankle angles and moments were shown in Fig. 3, GRFz and gluteus maximus were shown in Fig. 4, biceps femoris and vastus medialis were shown in Fig. 5, and medial gastrocnemius and tibialis anterior muscles were shown in Fig. 6. Significant pairwise differences and variable trends across conditions were summarized in Table 1 of Nordin and Dufek [1].
